# Prognostic value of negative stress cardiac magnetic resonance imaging in patients with moderate-severe coronary artery stenosis

**DOI:** 10.3389/fcvm.2023.1264374

**Published:** 2023-10-06

**Authors:** Ailís Ceara Haney, Janek Salatzki, Hauke Hund, Matthias G. Friedrich, Evangelos Giannitsis, Norbert Frey, Henning Steen, Dirk Loßnitzer, Johannes Riffel, Florian André

**Affiliations:** ^1^ Department of Cardiology, Angiology and Pneumology, Heidelberg University Hospital, Heidelberg, Germany; ^2^ German Centre for Cardiovascular Research (DZHK), Partner Site Heidelberg/Mannheim, Heidelberg, Germany; ^3^ GECKO Institute, Heilbronn University of Applied Sciences, Heilbronn, Germany; ^4^ Division of Cardiology, Departments of Medicine and Diagnostic Radiology, Mc-Gill University Health Centre, Montreal, QC, Canada; ^5^ First Department of Medicine-Cardiology, University Medical Centre Mannheim Mannheim, Germany; ^6^ German Centre for Cardiovascular Research (DZHK), Partner Site Heidelberg/Mannheim, Mannheim, Germany; ^7^ Division of Cardiology, Robert-Bosch-Medical Center Stuttgart, Stuttgart, Germany

**Keywords:** coronary artery disease, ischemia, outcome, cardiovascular imaging, stress cardiac magnetic resonance tomography

## Abstract

**Objective:**

This study aims to evaluate the prognostic value of stress cardiac magnetic resonance (CMR) without inducible ischemia in a real-world cohort of patients with known severe coronary artery stenosis.

**Background:**

The prognosis of patients with severe coronary artery stenosis and without inducible ischemia using stress CMR remains uncertain, even though its identification of functionally significant coronary artery disease (CAD) is excellent.

**Materials and methods:**

Patients without inducible ischemia and known CAD who underwent stress CMR between February 2015 and December 2016 were included in this retrospective study. These patients were divided into two groups: group 1 with stenosis of 50%–75% and group 2 with stenosis of >75%. The primary endpoint was defined as the occurrence of a major adverse cardiovascular event (MACE) [cardiac death, non-fatal myocardial infarction (MI), percutaneous coronary intervention (PCI), or coronary artery bypass grafting (CABG)].

**Results:**

Real-world data collected from 169 patients with a median age of 69 (60–75) years were included. The median follow-up was 5.5 (IQR 4.1–6.6) years. Events occurred after a mean time of 3.0 ± 2.2 years in group 1 and 3.7 ± 2.0 years in group 2 (*p* = 0.35). Sixteen (18.8%) patients in group 1 and 23 (27.4%) patients in group 2 suffered from MACE without a significant difference between the two groups (*p* = 0.33). In group 2, one cardiac death (1.2%), seven non-fatal MI (8.3%), 15 PCI (17.9%), and one CABG (1.2%) occurred.

**Conclusion:**

The findings of this pilot study suggest that long-term outcomes in a real-world patient cohort with known severe and moderate coronary artery stenosis but without inducible ischemia were similar. Stress CMR may provide valuable risk stratification in patients with angiographically significant but hemodynamically non-obstructive coronary lesions.

## Introduction

Patients with coronary artery disease (CAD) account for a significant burden of disease worldwide. Revascularization of coronary artery stenosis is recommended for severe obstruction of the vessel with a diameter of >90%, without the need for evidence of ischemia (1). Appropriate risk stratification of patients with a stenosis of unknown hemodynamic relevance and correct identification of those who may benefit from therapeutic intervention remain crucial. The current guidelines of the European Society of Cardiology for revascularization recommend that evidence of ischemia during a stress test is required to warrant revascularization for intermediate-grade stenosis, described as a visual assessment of stenosis of 40%–90% on invasive coronary angiography (ICA) (1). Moreover, evidence of ischemia detected through cardiac magnetic resonance (CMR) imaging indicates a worse prognosis such as cardiac death and non-fatal myocardial infarction (MI) (2, 3).

Stress testing using CMR imaging (stress CMR) shows a high sensitivity and specificity for detecting functionally relevant CAD compared with other imaging modalities (4–6). Perfusion imaging with adenosine or regadenoson is used to identify myocardial perfusion deficits revealing significant ischemia (7), whereas dobutamine stress CMR detects inducible wall motion abnormalities in patients with hemodynamically significant coronary lesions (6).

Previous studies have assessed the outcome of patients with suspected or known CAD and normal stress CMR and have demonstrated an excellent outcome in patients without inducible ischemia (8–10). However, these studies do not differentiate between patients with known CAD and those with suspected CAD. There is a lack of knowledge on the outcome of coronary artery stenosis without a hemodynamic significance differentiated by the severity of stenosis. Specifically, the outcomes of patients with severe stenosis and those with stenosis at the upper limit of the intermediate grade, without evidence of ischemia on stress CMR, remain unknown. To the best of our knowledge, this is the first study to assess the outcome of patients who have known CAD with moderate (50%–75%) to severe (>75%) stenosis without evidence of inducible ischemia on adenosine or dobutamine stress CMR.

## Materials and methods

### Study population

**Figure 1:**
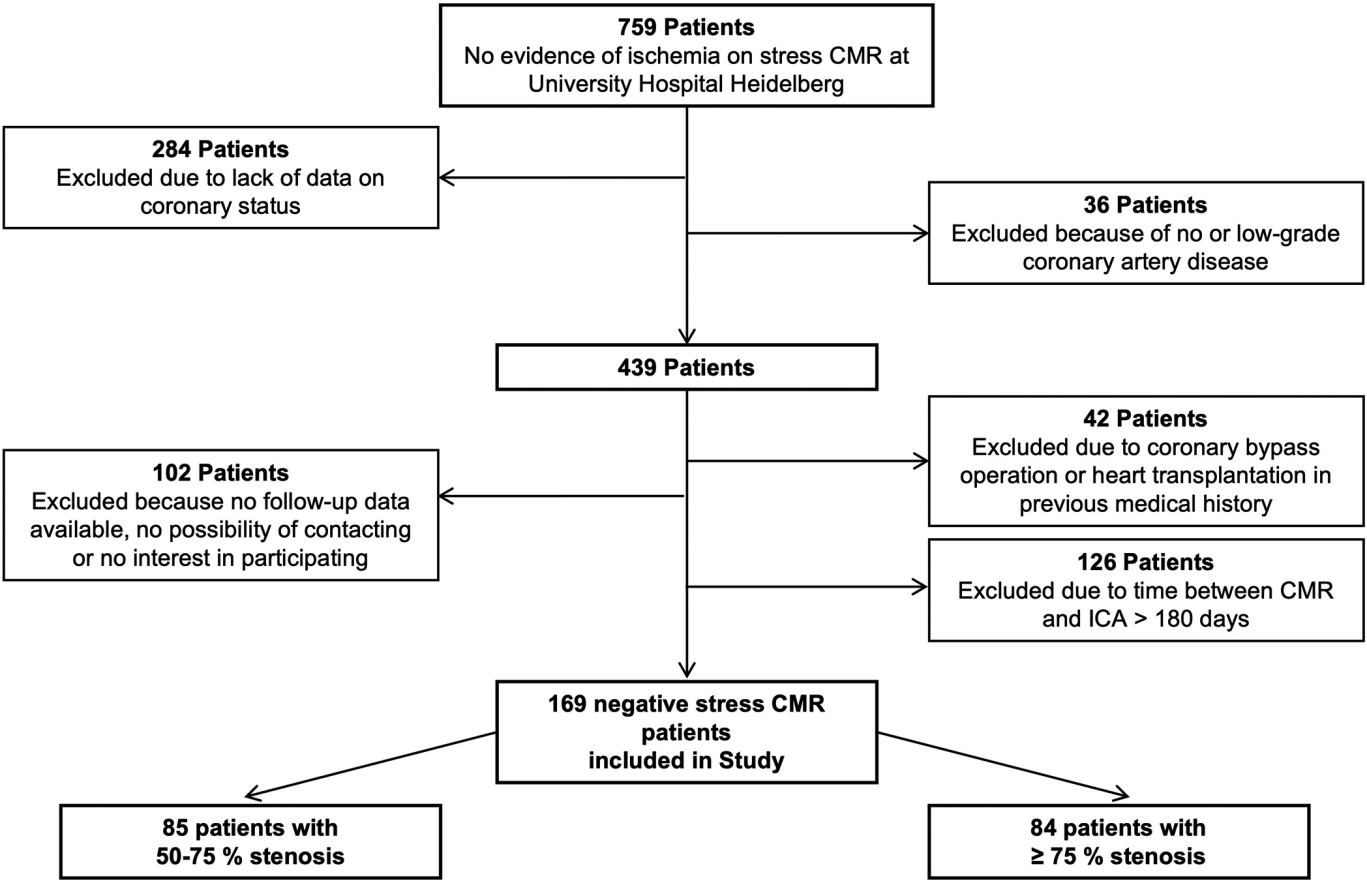
Flowchart of patient selection.

We conducted a longitudinal retrospective study by enrolling patients who had no inducible ischemia on adenosine or dobutamine stress CMR and who had known CAD with moderate (50%–75%) to severe (>75%) stenosis assessed by ICA or coronary computed tomography angiography (CTA). Patients with moderate stenosis were classified as group 1, and those with severe stenosis were classified as group 2. These patients underwent stress CMR examination 180 days before or after ICA at the Department of Cardiology, Angiology and Pneumology of the Heidelberg University Hospital between February 2015 and December 2016. Figure 1 presents a flowchart of how the patients are selected. ICA or CTA was performed to detect suspected obstructive CAD and MI. Obstructive CAD was defined as the visual diagnosis of >50% stenosis in the coronary arteries on ICA.

The reasons for performing dobutamine stress CMR were as follows: contraindications for adenosine stress CMR, such as renal insufficiency defined by an estimated glomerular filtration rate of <30 ml/min/1.73 m^2^, severe bronchoconstrictive or bronchospastic lung disease (e.g., asthma) and adenosine hypersensitivity, history of coronary artery bypass grafting (CABG) surgery, and chronic total or subtotal coronary occlusion (11).

A subgroup analysis was performed for patients with moderate and severe stenoses in the proximal segments of the coronary arteries on ICA or CTA. The proximal segments consisted of the proximal right coronary artery (P-RCA), left main (LM) coronary artery, proximal and middle left anterior descending artery (P-LAD and M-LAD), and proximal left circumflex artery (P-LCX). SYNTAX I score was calculated to determine the complexity of CAD using an online tool (https://syntaxscore.org) (12).

### Patient follow-up and outcome

The follow-up consisted of a clinical visit as part of usual care or by direct contact with the patient, and the follow-up time was at least 3 years. Data collection ended in October 2022. The primary combined endpoint was defined as the occurrence of a major adverse cardiovascular event (MACE), such as cardiac death, non-fatal MI, percutaneous coronary intervention (PCI), or CABG. Furthermore, a secondary combined endpoint that consisted of cardiac death and non-fatal MI was analyzed.

Cardiac death was defined as sudden cardiac death with documented fatal arrhythmias or death preceded by acute MI or acute or decompensated chronic heart failure. According to the fourth universal definition of acute MI, non-fatal MI was defined as typical angina lasting for more than 20 min, a rise in troponin above the 99th percentile, and ECG changes (13). In cases where several events occurred, the first event was counted, except for one patient who presented with non-ST-elevation MI (non-STEMI) who was treated with CABG 6 days later; here, CABG was counted.

### CMR protocol

#### CMR image analysis

A standard CMR was performed supine in a 1.5 T or 3 T full-body scanner (Ingenia CX and Ingenia, Philips Healthcare, Best, the Netherlands). Two-, three-, and four-chamber view long-axis cine and short-axis cine images covering the entire left ventricle (LV) from the base to the apex (8 mm slice thickness) were obtained using a breath-hold, balanced steady-state free precession sequence (bSSFP) with at least 35 phases per cardiac cycle. The data were analyzed using commercially available workstations (ViewForum™ and IntelliSpace™ Portal, ISPTM, Philips Healthcare, Best, the Netherlands) and a certified software (cmr42 Version 5.6.6, Circle Cardiovascular Imaging Inc., Calgary, Canada). LV end-diastolic (EDV), end-systolic volumes (ESV), LV ejection fraction (EF), and LV myocardial mass (LV mass) were obtained in short-axis stacks by semiautomatic manual tracing of epi- and endocardial borders, excluding the papillary muscles from the myocardium.

#### Adenosine perfusion stress CMR

In case of an inadequate heart rate response, stress perfusion imaging was performed using a continuous intravenous infusion of adenosine for at least 3 min at a rate of 140 μg/kg of body weight/min or 210 μg/kg of body weight/min. With three heartbeats after the sequence was initiated, a bolus of gadolinium–diethylenetriamine-pentaacetic acid (DTPA) (Magnevist™, Schering, Berlin, Germany) (before February 2016) or gadobutrol (Gadovist™, Bayer HealthCare, Leverkusen, Germany) (after February 2016) was injected over a separate peripheral venous catheter flushed with 20 ml of 0.9% saline solution at a rate of 5 ml/s. Myocardial perfusion imaging was performed in three LV short-axis slices (apical, mid-ventricular, and basal). Inducible ischemia was visually seen in the case of hypoperfusion of at least one AHA segment during adenosine perfusion, but it was not evident at baseline.

#### Dobutamine stress CMR

Dobutamine stress CMR was performed as previously described (11). Long-axis cine two-, three-, and four-chamber views and three short-axis views (apical, mid-ventricular, and basal) were obtained. Dobutamine was infused during the 3-min stages at incremental doses of 10, 20, 30, and 40 μg/kg of body weight/min until at least 85% of the age-predicted heart rate was reached (220 − age in years). Atropine was administered in 0.25 mg increments (up to a maximal dose of 2.0 mg) if the target heart rate was not reached. The images were assessed for wall motion abnormalities while at rest and at low, intermediate, and maximum stress. Electrocardiographic rhythm, symptoms, peripheral blood pressure, and oxygen saturation were continuously monitored during stress CMR.

Stress testing was stopped when the images were acquired at the target heart rate or when one of the following occurred: severe chest pain or dyspnea, severe arrhythmias, decrease in systolic blood pressure at >40 mmHg, arterial hypertension of >220/120 mmHg, and new or worsening wall motion abnormalities in at least one segment. CMR studies where patients did not achieve 85% of maximum age-predicted heart rate were considered non-diagnostic.

### Coronary angiography

The patients underwent ICA or CTA either for suspected obstructive CAD or for acute MI, such as ST-elevation MI (STEMI) and non-STEMI. Those with acute MI received revascularization therapy and were referred for stress CMR because of the remaining moderate-to-severe stenosis in other coronary artery segments. The examinations were visually analyzed by two experienced readers. The coronary artery segments were defined according to the current guidelines issued by the European Society of Cardiology (1).

### Statistical analysis

Statistical analysis was performed with MedCalc Version 20.114 (MedCalc Software, Ostend, Belgium). The Shapiro–Wilk test was used to determine the normal distribution of continuous variables, which were described as mean ± standard deviation or median and interquartile range (IQR), as appropriate. Variables with normal distribution were tested for statistical significance using the *t*-test. Variables without normal distribution were analyzed using the Mann–Whitney test and were presented as median and IQR. Categorical variables were compared using the chi-squared test. Kaplan–Meier curves were used for the visual representation of survival estimation as a function of follow-up time, and *p*-values were determined by performing log-rank testing as previously described (14). A *p*-value of <0.05 was considered statistically significant.

## Results

### Patient characteristics

We included 169 patients in the study (24% females), with a median age of 69 (IQR 60–75) years. A total of 85 (50.3%) patients had moderate stenosis (group 1), and 84 (49.7%) patients had severe stenosis (group 2). The baseline characteristics are presented in Table 1. Most patients underwent stress CMR after ICA. Twenty-four (14.2%) patients underwent ICA for acute MI, of which nine (5.3%) were for STEMI and 15 (8.9%) were for non-STEMI. The median time between ICA and subsequent stress CMR was 7 (2–97) days. Five (3.0%) patients underwent stress CMR before ICA. These five patients were referred for ICA after negative stress CMR due to persistent symptoms of angina. Another 19 (11.2%) patients underwent coronary CTA 87 ± 55 days before undergoing stress CMR. Approximately one-third in each coronary stenosis group underwent adenosine stress CMR [group 1, *n* = 29 (34.1%), vs. group 2, *n* = 24 (29.5%), respectively; *p* = 0.55].

**Table 1:** Baseline characteristics of patients with moderate and severe coronary artery stenoses.

Patient characteristics	Moderate stenoses (*n* = 85)	Severe stenoses (*n* = 84)	*p*-value
Age, years	68.0 (58.0–75.0)	70.0 (62.0–76.0)	0.17
Female, *n* (%)	22 (25.3)	19 (23.2)	0.62
BMI (kg/m^2^)	26.7 ± 4.3	26.9 (24.8–29.7)	0.40
Cardiovascular RF			
Hypertension, *n* (%)	74 (87.1)	72 (85.7)	0.80
Hypercholesterinemia, *n* (%)	67 (78.8)	67 (79.8)	0.88
Smoking, *n* (%)	33 (38.8)	28 (33.3)	0.46
Diabetes, *n* (%)	26 (30.1)	23 (27.4)	0.65
Positive family history, *n* (%)	22 (25.9)	37 (44.0)	0.01
STEMI at initial presentation, *n* (%)	1 (1.2)	8 (9.5)	0.02
Non-STEMI at initial presentation, *n* (%)	5 (5.9)	10 (11.9)	0.16
Adenosine, *n* (%)	29 (34.1)	25 (29.8)	0.55
Dobutamine, *n* (%)	56 (65.9)	59 (70.2)	0.55
EF, %	57.3 (51.3–62.3)	59.9 (56.0–63.5)	0.06
EDV, ml	157.0 ± 38.4	143.5 (126.5–178.0)	0.34
ESV, ml	68.8 (52.0–84.0)	67.0 (47.0–77.5)	0.11
LV mass, g	98.8 ± 24.9	99.0 (82.0–118.0)	0.61
Location of coronary artery stenosis, *n* (%)			
LM	8 (9.4)	1 (1.2)	
LAD, proximal	25 (29.4)	4 (4.8)	
LAD, middle	33 (38.8)	8 (9.5)	
LAD, distal	18 (21.1)	8 (9.5)	
LAD, first D	23 (27.1)	23 (27.4)	
LAD, second D	6 (7.1)	8 (9.5)	
LCX, proximal	5 (5.9)	3 (3.6)	
LCX, obtuse marginal	14 (16.5)	21 (25.0)	
LCX, distal	13 (15.3)	10 (11.9)	
LCX, left posterolateral	5 (5.9)	8 (9.5)	
LCX, left posterior descending	5 (5.9)	15 (17.9)	
RCA, proximal	9 (10.1)	7 (8.3)	
RCA, middle	13 (15.3)	6 (7.1)	
RCA, distal	9 (10.1)	15 (17.9)	
RCA, right posterior descending	6 (7.1)	16 (19.0)	
SYNTAX I score	9 (5–14.25)	14 (9–20)	0.0002

BMI, body mass index; D, diagonal branch; EDV, end-diastolic volume; EF, ejection fraction; ESV, end-systolic volume; LAD, left anterior descending artery; LCX, left circumflex artery; LM, left main coronary artery; LV, left ventricle; non-STEMI, non-ST-elevation myocardial infarction; RCA, right coronary artery; STEMI, ST-elevation myocardial infarction; SYNTAX, SYNergy between PCI with TAXUS and cardiac surgery.

Characteristics of patients with moderate coronary artery stenoses compared with patients with severe coronary artery stenoses. Values are mean ± SD, median (interquartile range), or *n* (%). Differences between groups were calculated using the *t*-test, Mann–Whitney *U* test, or chi-squared test.

The study population showed a significant number of patients with cardiovascular risk factors, predominantly hypertension and hypercholesterolemia. No significant difference was found in medication during follow-up between patients with moderate stenosis and those with severe stenosis, specifically not concerning treatment with antiplatelet therapy and cholesterol-lowering drugs (see Supplementary Material Table S1). Patients being treated for STEMI at initial presentation had residual severe stenosis significantly more often than residual moderate stenosis [*n* = 8 (9.5%) vs. *n* = 1 (1.2%) respectively; *p* = 0.02] (Table 1).

No significant difference in LVEF, LVEDV, LVESV, and LV mass was reported between the two groups (Table 1). The median LVEF was preserved with 57.3% (IQR 51.3%–62.3%) and 59.9% (IQR 56.0%–63.5%) in group 1 and group 2, respectively. A total of 52 patients (30.7%) demonstrated subendocardial late gadolinium enhancement (LGE) on CMR.

Among patients in group 2, severe stenoses were identified in proximal segments in 20 (23.8%) patients and in distal segments in 64 (76.2%) patients. Thirty-nine (23.1%) patients showed a diameter stenosis of >90%. SYNTAX I scores differed significantly between patients with moderate stenoses (nine, IQR 5–14.25) and patients with severe stenoses (IQR 14–20) (Table 1).

### Clinical endpoints and outcomes

**Figure 2:**
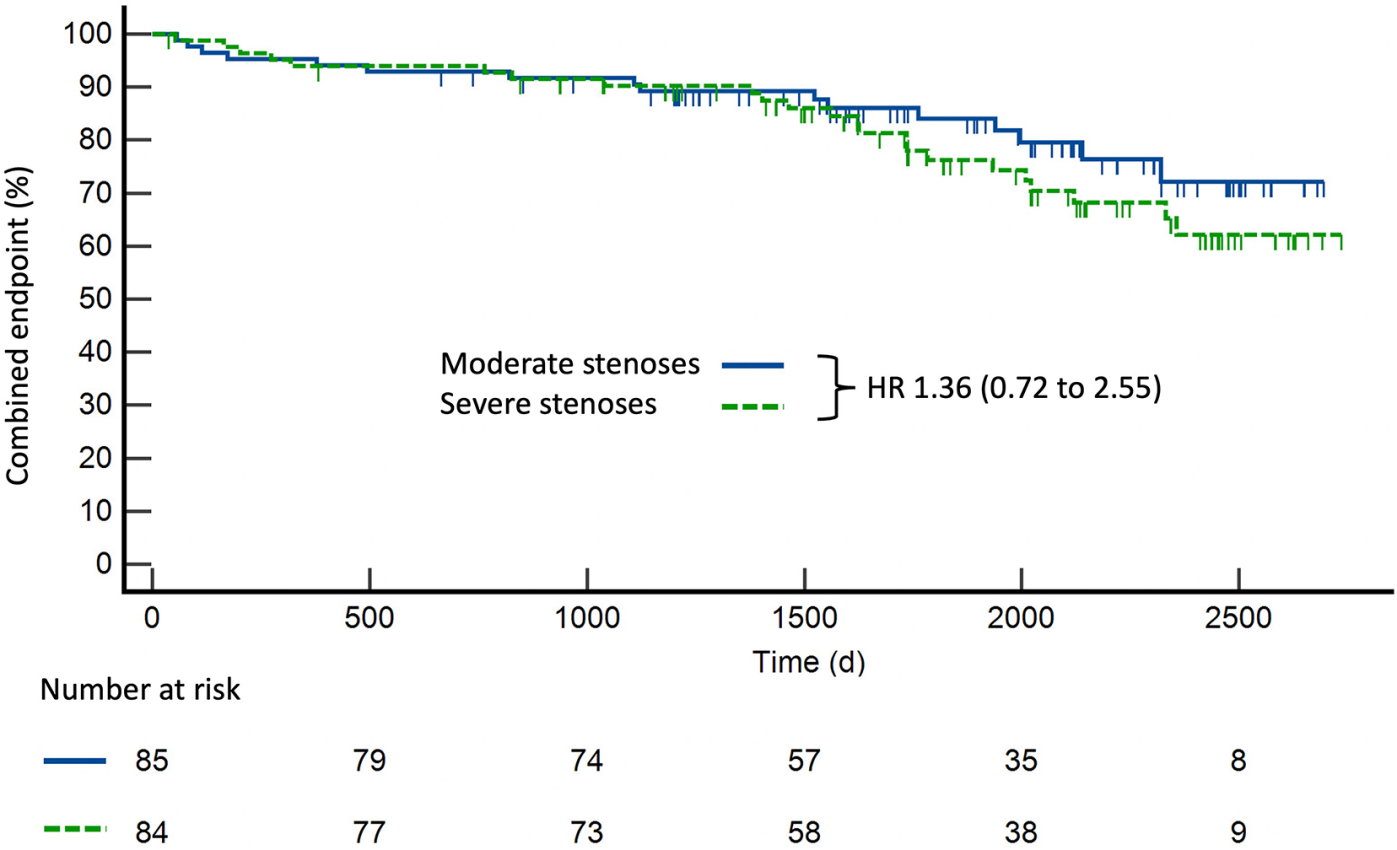
Kaplan–Meier curve for survival of patients with moderate coronary artery stenoses compared with patients with severe coronary artery stenoses.

After a median follow-up of 5.5 (IQR 4.1–6.6) years, the primary combined endpoint occurred in 16 (18.8%) patients with moderate stenosis and 23 (27.4%) patients with severe stenosis (HR 1.36, CI 0.73–2.56, *p* = 0.33), showing no significant differences between the two groups (Figure 2). MACE was primarily accountable to PCI, with 13 (15.3%) patients with moderate stenosis and 15 (17.9%) patients with severe stenosis receiving elective PCI (HR 1.09, CI 0.52–2.30, *p* = 0.81), due to unstable angina (Table 2). Acute MI occurred in three (3.5%) patients with moderate stenosis and eight patients with severe stenosis (9.5%) (HR 2.36, CI 0.72–7.70, *p* = 0.16). Excluding revascularization by PCI or CABG from the primary combined endpoint, we found no significant difference in the outcomes between the two groups (HR 2.34, CI 0.72–7.66, *p* = 0.16).

**Table 2:** Clinical endpoints reached by patients with moderate and severe coronary artery stenoses.

Clinical endpoint	Moderate stenoses (*n* = 85)	Severe stenoses (*n* = 84)	HR (CI)
MACE, *n* (%)	16 (18.8)	23 (27.4)	1.36 (0.73–2.56)
Cardiac death, *n* (%)	0 (0.0)	1 (1.2)	
ACS, (%)	3 (3.5)	7 (8.3)	2.36 (0.72–7.70)
STEMI, *n* (%)	0 (0.0)	1 (1.2)	
Non-STEMI, *n* (%)	3 (3.5)	6 (7.1)	1.82 (0.49–6.75)
PCI, *n* (%)	13 (15.3)	15 (17.9)	1.09 (0.52–2.30)
CABG, *n* (%)	0 (0.0)	1 (1.2)	
Non-cardiac death, *n* (%)	2 (2.4)	7 (8.3)	2.92 (0.79–10.81)
All-cause death, *n* (%)	2 (2.4)	8 (9.5)	3.18 (0.92–11.00)

ACS, acute coronary syndrome; CABG, coronary artery bypass graft; MACE, major adverse cardiovascular events; non-STEMI, non-ST-elevation myocardial infarction; PCI, percutaneous coronary intervention; STEMI, ST-elevation myocardial infarction.

Clinical endpoints reached by patients with moderate coronary artery stenoses compared with patients with severe coronary artery stenoses. Values are mean ± SD, median (interquartile range), or *n* (%). Differences between groups were calculated using the *t*-test, Mann–Whitney *U* test or chi-squared test.

All-cause death occurred in two (2.4%) patients with moderate stenosis and in eight (9.5%) patients with severe stenosis (HR 3.18, CI 0.92–11.0, *p* = 0.07). No cardiac death was recorded in patients with moderate stenosis, compared with one cardiac death (1.2%) in patients with severe stenosis. Arrhythmic events such as atrioventricular block or pulmonary vein isolation were rare during follow-up (Supplementary Material Table S3). These events occurred after a mean time of 3.0 ± 2.2 years in patients with moderate stenosis and after a mean time of 3.7 ± 2.0 in patients with severe stenosis (*p* = 0.35). The Kaplan–Meier curve for the primary combined endpoint started to separate after approximately 270 days. In patients with severe coronary stenosis, the probability of an event-free period within the first 9 months was found at 95%. Similarly, within the first 12 months after stress CMR, the probability of an event-free period was at 94%.

**Figure 3:**
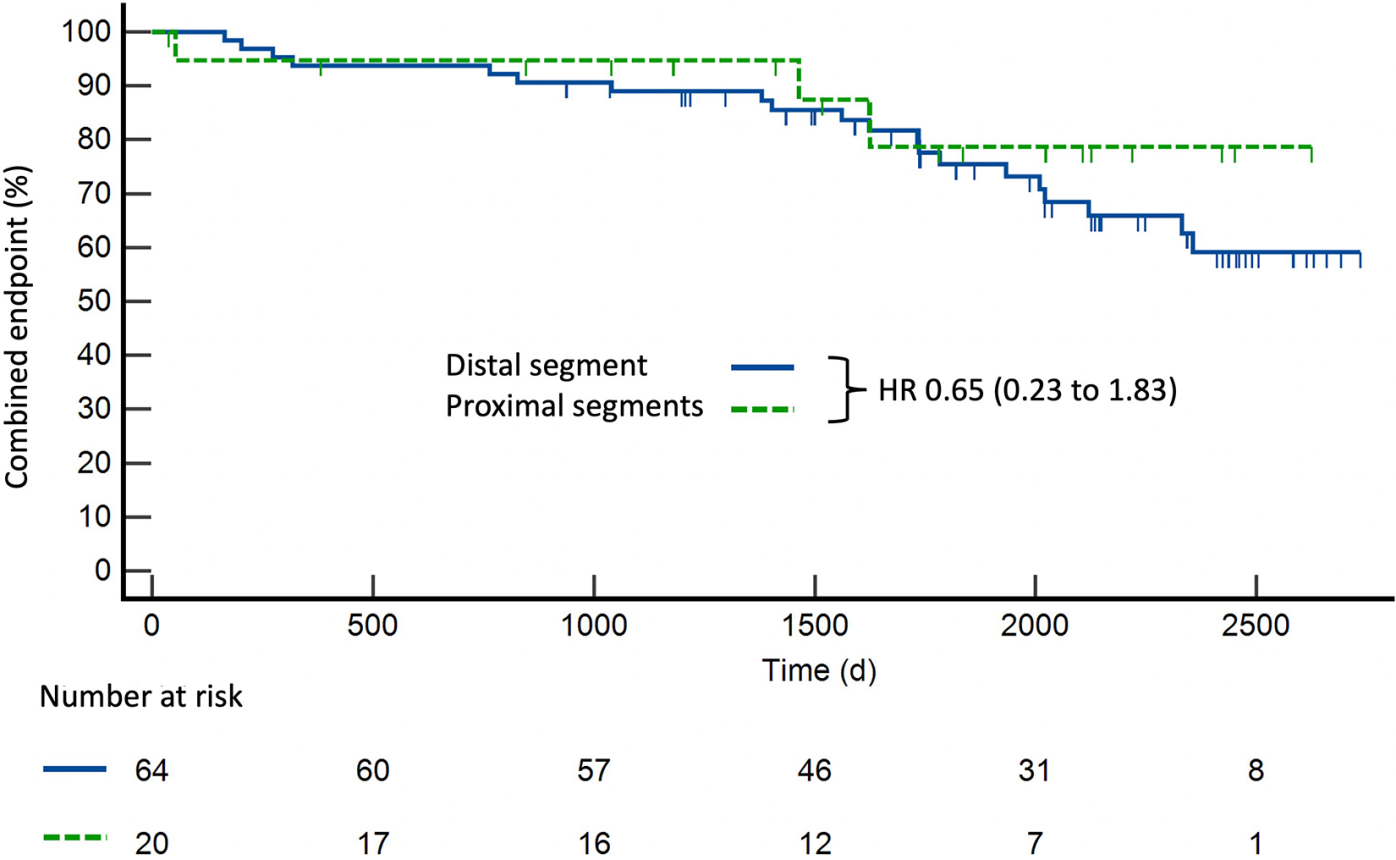
Kaplan–Meier curve for survival of patients with severe coronary artery stenoses in proximal segments (*n* = 20) compared with distal segments (*n* = 64). Proximal segments were defined as the proximal right coronary artery, left main coronary artery, proximal and middle left anterior descending artery, and proximal left circumflex artery.

In a subgroup analysis of patients with severe lesions, no significant difference was noted in outcomes between patients with proximal stenosis and those with distal stenosis (HR 0.65, CI 0.23–1.83, *p* = 0.42) (Figure 3). In addition, a subgroup analysis was performed on patients with a stenosis of >90% compared with those with a stenosis of <90%. The outcome was similar between these two groups (HR 1.12, CI 0.56–2.25, *p* = 0.75). There was also no significant difference in the outcomes between patients with subendocardial LGE and those without LGE (HR 0.90, CI 0.46–1.79, *p* = 0.77). The outcome did not differ significantly between patients in whom stress CMR was performed with adenosine and in those in whom stress CMR was performed with dobutamine (HR 0.74, CI 0.38–1.43, *p* = 0.37). The extent of symptoms during follow-up did not differ significantly between the two groups. Specifically, 12 patients with moderate stenoses and 11 patients with severe stenoses suffered from angina (19.0% and 15.3%, respectively, with a *p*-value of 0.56), while 15 patients with moderate stenoses and 12 patients with severe stenoses had dyspnea (23.8% and 16.7%, respectively, with a *p*-value of 0.30) (Supplementary Material Table S2).

### Individual outcomes

Patients who reached the endpoint of cardiac death, MI, or CABG were followed up individually (Table 3). Twelve patients reached one of these endpoints. The mean time between CMR and endpoint was 3.6 ± 2.1 years. In five patients (patients 2, 3, 6, 8, and 11) (41.6%) who presented with MI, the evaluation of their coronary artery segments in stress CMR was causal for the event. In one (patient 6) out of these five patients, the initial lesion was severe, and in four patients (patients 2, 3, 8, and 11), the initially evaluated lesion was moderate. One patient (patient 10) who presented with non-STEMI 3 years after negative stress CMR to evaluate moderate-to-severe LCX stenosis showed a relevant progression of CAD with severe stenoses in proximal LAD and LCX, warranting urgent CABG. Two patients (patients 1 and 4) who presented with MI that was not caused by coronary artery lesions were initially evaluated by stress CMR. Patient 9 revealed no significant progression of CAD on ICA. Thromboembolism was suspected as the cause of non-STEMI because the patient on his own had recently discontinued oral anticoagulation that had been initiated for deep vein thrombosis without an apparent cause 3 years earlier and residual thrombosis in follow-up exams afterward.

**Table 3:** Individual follow-up for patients who reached the endpoint cardiac death, ACS, or CABG.

Patient	Coronary artery segment assessed by CMR	Event	Coronary artery segment causal of event	Additional information
1	LAD 8 75% LAD 9 90%	STEMI	LCX (RIM)	In-stent-stenosis after non-STEMI RIM
2	LAD 8 75% LAD 6 50–75% LCX 12 75% RCA 1 50%	Non-STEMI	LAD 6	Followed by PCI/2DES RCA, PCI/3DES LAD, cardiac death due to non-STEMI without ICA
3	LAD 7 50% LCX 12 50%	Non-STEMI	LAD 6 + 7	
4	LAD 7 + 8 50% LAD 9 90% LCX 13 + 15 50% LCX 14 90% RCA 1 50%	Non-STEMI	LCX 12	
5	RCA 1 50% LM 50%	Non-STEMI	–	No PCI, palliative care due to advanced age, apical akinesia in echo
6	LAD 6 50% LAD 9 90% LAD 10 99% LCX 15 100%	Non-STEMI	LAD 6 (2x DES), LAD 9 (DEB)	
7	LAD 9 99% LCX 12 90% RCA 4 90%	Non-STEMI	LCX 11	
8	LAD 6 + 7 50%	Non-STEMI	LAD 7	
9	LAD 7 + 8 50% RCA 4 100%	MINOCA	–	
10	LCX 13 50% LCX 15 75%	CABG	LAD, LCX	Initial presentation: non-STEMI without intervention, CABG 6 days later
11	RCA 1 75% RCA 2 50% LAD 6 75% LCX 11 + 13 50%	Non-STEMI	LCX 11–13	
12	LAD 9 75%	Cardiac death	–	Asystole, cardiogenic shock

ACS, acute coronary syndrome; CABG, coronary artery bypass graft; CMR, cardiac magnetic resonance imaging; DEB, drug-eluting balloon dilatation; DES, drug-eluting stent implantation; non-STEMI, non-ST-elevation myocardial infarction; PCI, percutaneous coronary intervention; STEMI, ST-elevation myocardial infarction; LM, left main coronary artery; LAD, left anterior descending artery (proximal, 6; middle, 7; distal, 8; first diagonal branch, 9; second diagonal branch, 10). LCX, left circumflex artery (proximal, 11; obtuse marginal branch, 12, distal, 13; left posterolateral, 14; left posterior descending, 15; RIM, ramus intermedius artery). RCA, right coronary artery (proximal, 1; middle, 2; distal, 3; right posterior descending, 4).

Red indicates patients in whom the initially evaluated severe lesion was causal for the event. Blue indicates patients in whom the initially evaluated moderate lesion was causal for the event.

Two patients with non-STEMI were not evaluated by ICA. The first patient (patient 12) presented with cardiogenic shock and suffered from cardiac arrest due to asystole before ICA could be performed. In the second patient (patient 5), palliative care was decided due to old age and progressive respiratory failure caused by pneumonia.

## Discussion

The findings in our study indicated the following: (1) in patients with a confirmed diagnosis of CAD, yet without any signs of inducible ischemia on stress CMR, the rate of occurrence of hard cardiac events and the need for revascularization are exceedingly low, irrespective of the severity of coronary artery stenosis; (2) no significant difference in the rate of MACE occurrence between patients with severe stenosis in proximal coronary artery segments and those with stenosis in distal segments was found; (3) in the majority of patients experiencing a hard cardiac event or requiring revascularization, it was found that the initially evaluated coronary lesion was not responsible for such an event; and (4) in patients with severe coronary artery stenosis, the likelihood of their experiencing a hard cardiac event within the first 9 months after stress CMR, without inducible ischemia, was found to be below 5%. Furthermore, this probability slightly increased to approximately 6% within the first 12 months.

To the best of our knowledge, this study in a real-world patient cohort represents the first investigation of the long-term outcomes of severe coronary artery stenosis without a functional significance using stress CMR. In previous studies that examined the prognostic value of stress CMR, patients with both known and suspected CAD were included, with no differentiation between the two groups of patients. Oftentimes, known CAD is defined as a history of PCI, MI, or CABG, which fails to provide data on residual stenoses and the severity of these stenoses (9, 15).

A notable strength of our study is the patient cohort that we investigated: CMR was performed as part of standard clinical practice, and the patients were not subjected to specific preselection for inclusion in our analysis, making the data representative of a real-world patient population. Our examination focused on patients exhibiting a substantial prevalence of cardiovascular risk factors, suggesting a population characterized by a significant burden of illness. This is also reflected in the choice of the stress agent: A significant number of patients were stressed with dobutamine due to comorbidities such as renal insufficiency or chronic total coronary occlusion.

The primary combined endpoint in our study was mainly driven by the occurrence of PCI. It is important to note that in our study, MACE was specifically focused on the occurrence of cardiac events, excluding stroke or all-cause death. The overall outcome was favorable. This is in line with the results from a randomized controlled trial presented by Doesch et al., which demonstrated a favorable prognosis of patients with intermediate coronary artery lesions, which were specifically defined as stenosis ranging from 50% to 75%, without perfusion deficit on adenosine stress CMR (16). In addition, our study results were consistent with the findings of the DEFER study, which also exhibited an excellent 5-year outcome in patients with stable chest pain and angiographically significant coronary artery stenoses but without evidence of inducible ischemia through non-invasive testing (17).

In our study, it was observed that in the majority of cases where patients suffered from MI, cardiac death, or CABG, the initially evaluated coronary artery lesion was not found to be the underlying cause of the event. In five patients, the initially evaluated lesion was responsible for hard cardiac events. However, the lesion was severe in only two patients. These findings aligned with the results of the ICONIC trial, which focused on patients undergoing CTA in a nested case–control trial to identify the precursors of acute coronary syndrome (ACS) (18). Most patients enrolled in the ICONIC trial did not show severe stenoses before experiencing ACS. This highlights the fact that atherosclerotic features and composition of plaques, particularly the presence of high-risk plaques, are more important than the severity of the lesions in predicting high-risk patients for ACS.

Similarly, in the extended DEFER trial, which utilized fractional flow reserve measurement to assess functionally non-significant coronary artery lesions, during follow-up, only one MI was possibly related to the vessel evaluated (19).

Our study supports a previous finding indicating that the visual assessment of stenosis grade by angiography does not solely determine the outcomes in patients with CAD. The hemodynamic significance of coronary stenosis correlates poorly with a visual assessment of ICA (20). This discrepancy was evident in the FAME trial. In this trial, approximately one-third of moderate stenoses, between 50% and 70%, were hemodynamically significant, and one-fifth of severe stenoses, between 70% and 90%, were not (21). Therefore, stress CMR had proved excellent in identifying functional CAD. Patients with evidence of ischemia on stress CMR had a worse prognosis, such as a higher risk for cardiac death and non-fatal MI (2, 3). Whether or not revascularization reduces the risk of mortality for patients with chronic coronary syndrome and ischemia remains a topic of current research (6, 22, 23).

Our study also supports the notion that stress CMR is a clinically valuable technique in guiding revascularization in patients with CAD, even in patients with angiographically known severe coronary artery stenosis.

### Limitations

We acknowledge several limitations in our study. First, this study might have been underpowered due to the limited number of cases. Unfortunately, due to the absence of complete follow-up data in both groups, the size of the study cohort remained relatively small. Coupled with the retrospective design of our study, these factors allowed us only to hypothesize rather than conclude that patients with myocardial ischemia demonstrated a low rate of cardiac events even with known severe coronary artery stenoses on ICA. Furthermore, the severity of stenosis was assessed visually on coronary angiography performed by two experienced cardiologists, without using an intravascular ultrasound or optical coherence tomography. It is a well-known fact that there is a significant inter- and intraobserver variability in the visual assessment of the severity of coronary artery stenosis on ICA (24, 25). In addition, the hemodynamic significance of the stenosis was solely assessed non-invasively and not with fractional flow reserve measurement. Moreover, our cohort study on the subject is smaller than that of other studies. We included patients who underwent dobutamine and adenosine stress CMR, which are two different agents and vary in sensitivity and specificity in detecting functionally significant CAD. In future studies, a single stress agent is recommended. In addition, previous studies demonstrated that a quantitative perfusion analysis of ischemia performed better than a visual assessment of adenosine perfusion images in terms of the prognostic value in patients with suspected CAD (26, 27). Quantitative perfusion measurement was not performed because of the retrospective nature of this study. It may also improve the prognosis in patients with known severe coronary artery stenoses.

As indicated by the comparable incidence of clinical symptoms, patients with moderate coronary artery stenoses are affected by coronary microvascular dysfunction (CMD). Using a CMR perfusion measurement while both at rest and during adenosine stress, myocardial perfusion reserve index (MPRI) can be measured for evaluating CMD (28, 29). Previously, MPRI was found to be an independent prognostic marker for MACE among patients who experienced chest pain (28). However, perfusion measurement while at rest is not standard practice in stress CMR during regular clinical scans. Therefore, MPRI could not be calculated. Additional studies are needed to investigate the impact of CMD on symptom relief in patients with concurrent CAD.

Furthermore, the main aim of our study was to assess the prognostic value of negative stress CMR. Hence, a control group of matched patients with positive stress CMR was beyond the scope of our research. Moreover, the follow-up process may be biased by self-reporting bias as it involves direct contact with patients via telephone. Finally, it is important to note that our study design was retrospective, resulting in limitations in terms of data collection and potential biases associated with retrospective analyses.

## Conclusion

Our study suggests that patients with negative stress CMR may have a good prognosis irrespective of the severity of angiographic coronary stenosis. The likelihood of suffering from MACE or requiring revascularization within the first 12 months after a negative stress CMR is remarkably low, even in patients with severe coronary stenosis on angiography. Stress CMR, using adenosine or dobutamine stress agents, may be a valuable risk stratification tool for patients with CAD. Additional prospective studies with larger cohorts of patients with severe coronary stenoses are required to prove our findings.

## Data Availability

The raw data supporting the conclusions of this article will be made available by the authors, without undue reservation.
