# Supplementary material for: Prognostic value of negative stress cardiac magnetic resonance imaging in patients with moderate-severe coronary artery stenosis
Source: Front Cardiovasc Med. 2023 Oct 6;10:1264374. doi: 10.3389/fcvm.2023.1264374 (PMC10588178; doi:10.3389/fcvm.2023.1264374)
Supplement: Supplementary file 1 [file Datasheet1.docx]

**Table 1:** Medications of patients with moderate and severe coronary artery stenoses. Data in 14 patients with moderate coronary artery stenoses and in 10 patients with severe coronary artery stenoses were not available.

| Medications | Moderate stenoses  (n = 71) | Severe stenoses  (n = 74) | p-value |
| --- | --- | --- | --- |
| Aspirin, n (%) | 48 (68) | 46 (63) | 0.494 |
| P2Y12 inhibitor, n (%) | 10 (14) | 18 (24) | 0.200 |
| Direct factor X1 inhibitor, n (%) | 18 (25) | 25 (34) | 0.268 |
| Phenprocoumon, n (%) | 1 (1) | 1 (1) | 0.977 |
|  |  |  |  |
| Statin, n (%) | 62 (87) | 67 (91) | 0.538 |
| Ezetimibe, n (%) | 23 (32) | 24 (32) | 0.996 |
| Bempedoic acid, n (%) | 2 (3) | 4 (5) | 0.436 |
| PCSK9 inhibitor, n (%) | 0 (0) | 1 (1) | 0.426 |
| Inclisiran, n (%) | 1 (1) | 0 (0) | 0.426 |
|  |  |  |  |
| Ranolazine, n (%) | 6 (7) | 5 (6) | 0.700 |
| Molsidomine, n (%) | 2 (2) | 1 (1) | 0.537 |
|  |  |  |  |
| Beta-blocker, n (%) | 46 (54) | 53 (63) | 0.379 |
| ACE inhibitor, n (%) | 21 (25) | 24 (29) | 0.711 |
| AT1 antagonist, n (%) | 30 (35) | 32 (38) | 0.905 |
| Aldosterone receptor antagonist, n (%) | 9 (13) | 16 (22) | 0.155 |
| Angiotension receptor-neprilysin inhibitor, n (%) | 1 (1) | 6 (7) | 0.061 |
| SGLT2 inhibitor, n (%) | 10 (12) | 14 (17) | 0.435 |
| Diuretic, n (%) | 17 (20) | 27 (32) | 0.102 |
|  |  |  |  |
| Calcium channel blocker, n (%) | 17 (20) | 23 (27) | 0.336 |
| Moxonidine, n (%) | 1 (1) | 3 (4) | 0.333 |
| Aliskiren, n (%) | 0 (0) | 2 (2) | 0.165 |
| Doxazosin, n (%) | 1 (1) | 2 (2) | 0.584 |
|  |  |  |  |
| Amiodarone, n (%) | 2 (3) | 3 (4) | 0.684 |
| Digitoxin, n (%) | 1 (1) | 1 (1) | 0.977 |
| Flecainide, n (%) | 1 (1) | 0 (0) | 0.426 |
|  |  |  |  |

Medications of patients with moderate coronary artery stenoses compared to patients with severe coronary artery stenoses. Differences between groups were calculated using chi-squared test.

**Table 2:** Symptoms during follow-up of patients with moderate and severe coronary artery stenoses. Follow up on symptoms was not available for 34 patients (20.1 %).

|  | Moderate stenoses  (n = 63) | Severe stenoses  (n = 72) | p-value |
| --- | --- | --- | --- |
| Angina, n (%) | 12 (19.0) | 11 (15.3) | 0.56 |
| Dyspnea, n (%) | 15 (23.8) | 12 (16.7) | 0.30 |
| Dizziness, n (%) | 6 (9.5) | 5 (6.9) | 0.57 |
| Syncope, n (%) | 2 (3.2) | 1 (1.4) | 0.48 |

**Table 3:** Arrhythmic events during follow-up of patients with moderate and severe coronary artery stenoses. Follow up on symptoms was not available for 32 patients (18.9 %).

|  | Moderate stenoses  (n = 64) | Severe stenoses  (n = 73) | p-value |
| --- | --- | --- | --- |
| Ventricular tachycardia, n (%) | 0 | 1 (1.4) | 0.35 |
| Ablation of ventricular ectopic beats, n (%) | 0 | 1 (1.4) | 0.35 |
| Pacemaker implantation, n (%) | 2 (3.1) | 1 (1.4) | 0.49 |
| ICD implantation, n (%) | 0 | 1 (1.4) | 0.35 |
| Pulmonary vein isolation, n (%) | 4 (6.2) | 4 (5.5) | 0.85 |
| SA block, n (%) | 3 (4.7) | 1 (1.4) | 0.25 |
| AV block, n (%) | 2 (3.1) | 2 (2.7) | 0.89 |

Arrhythmic events of patients with moderate coronary artery stenoses compared to patients with severe coronary artery stenoses. ICD – implantable cardioverter defibrillator. SA – sinoatrial. AV – atrioventricular.
